# Characterisation of a novel paralog of scavenger receptor class B member I (*SCARB1*) in Atlantic salmon (*Salmo salar*)

**DOI:** 10.1186/1471-2156-12-52

**Published:** 2011-05-30

**Authors:** Hilde Sundvold, Hanna Helgeland, Matthew Baranski, Stig W Omholt, Dag Inge Våge

**Affiliations:** 1 Centre for Integrative Genetics, Dept. of Animal and Aquacultural Sciences, Norwegian University of Life Sciences, 1432 Aas, Norway; 2 Nofima Marin, 1432 Aas, Norway

## Abstract

**Background:**

Red flesh colour is a unique trait found in some salmonid genera. Carotenoid pigments are not synthesized *de novo *in the fish, but are provided by dietary uptake. A better understanding of the molecular mechanisms underlying the cellular uptake and deposition of carotenoids could potentially be used to improve the low muscle deposition rate that is typically found in farmed Atlantic salmon. In addition, from an evolutionary point of view, the establishment and maintenance of this trait is still poorly understood. It has been demonstrated in several species that scavenger receptor class B, member 1 (*SCARB1*) is involved in intestinal absorption of carotenoids, which makes this gene a possible source of genetic variation in salmonid flesh pigmentation.

**Results:**

In this study, a novel paralog of *SCARB1 (SCARB1-2) *was detected through screening for genetic variation in Atlantic salmon *SCARB1*. Full length *SCARB1-2 *encodes a protein with 89% identity to Atlantic salmon *SCARB1*, except for the C-terminal cytoplasmic tail that shows only 12% identity. The most prominent site of *SCARB1 *mRNA expression was in the mid gut, while a five-fold lower level was detected in Atlantic salmon skeletal muscle and liver. The *SCARB1-2 *mRNA was equally expressed in liver, muscle and mid gut, and at a lower level than *SCARB1 *mRNA. A total of seven different *SCARB1-2 *alleles comprising repetitive enhancer of zeste motifs (EZH2) were identified in the founding parents of a resource Atlantic salmon population. We mapped the *SCARB1-2 *paralog to a region on Atlantic salmon chromosome 1, containing a putative QTL for flesh colour. Addition of the *SCARB1-2 *marker increased the significance of this QTL, however the large confidence interval surrounding the QTL precludes confirmation of *SCARB1-2 *as a causative gene underlying variation in this trait.

**Conclusion:**

We have characterised a novel paralog of *SCARB1 (SCARB1-2)*, have mapped it to Atlantic salmon chromosome 1 and have described its expression in various tissues. Mapping with *SCARB1-2 *alleles added further evidence for a QTL affecting flesh colour on this chromosome, however further studies are needed to confirm a functional role for this gene in flesh colour pigmentation.

## Background

The most prominent quality characteristic of Atlantic salmon is the red/pink flesh colour that is caused by accumulation of carotenoids in the muscle [1]. Since no *de novo *synthesis of carotenoids occur *in vivo*, dietary supplied astaxanthin serves as the main source of carotenoids in farmed Atlantic salmon. Astaxanthin supplementation represents a considerable cost for the aquaculture industry [1], and the absorption and muscle deposition efficiency is generally low [2,3]. Low to medium heritability estimates (h^2 ^mean = 0.3) for flesh colour have been calculated for various salmonids (reviewed in [4]). An improved understanding of the genetic component of this trait and the molecular mechanisms involved could pave the way for more targeted selection for improved flesh pigmentation. Besides the commercial interest in this trait, the evolutionary basis of carotenoid deposition in the muscle of salmonids is still not well understood [5].

The processes involved in the intestinal uptake and transport of carotenoids in the blood serum are closely linked with that of fatty acids, and seem to be common in most carotenoid pigment-containing vertebrates [6-8]. Upon digestion, carotenoids are incorporated into mixed micelles [9], and carotenoid esters are hydrolyzed by bile salt-stimulated lipase [10] prior to absorption in the intestine. A fraction of the provitamin A carotenoids like beta-carotene are converted to vitamin A, while the rest are reassembled together with fatty acids into chylomicrons that enter the blood and are transported in the circulation. Besides providing vitamin A [11,12], astaxanthin serves as a very potent antioxidant, effectively quenching singlet oxygen, scavenging free radicals, and preventing lipid peroxidation [13,14]. Astaxanthin is transported by the High Density Lipoprotein (HDL) and the Very High Density Lipoprotein (VHDL) fractions of the plasma of chum salmon, *Oncorhynchus keta *[15], and high levels of astaxanthin are observed in the plasma of salmonids [16]. Carotenoid accumulation in various tissues is observed in a wide range of animals and fish at varying levels [17,18].

One approach applied for identification of genes contributing to complex traits is through screening for variation in biologically relevant candidate genes and analysing whether any of these are linked to the trait of interest. It has been suggested that flesh pigmentation in fish is controlled by a small number of genes with large non-additive effects [19]. Despite a scarce knowledge of molecular mechanisms involved in carotenoid flesh deposition in fish, Rajasingh *et al.*, [5] predicted by mathematical modelling that the rate of carotenoid uptake into the muscle tissue, together with the intestinal uptake, have the highest influence on actual muscle pigment concentrations. Animal studies have identified an important role of the scavenger receptor class B, type 1 (*SCARB1*), in the intestinal absorption of dietary lipids [20]. In Atlantic salmon, a particularly high *SCARB1 *mRNA expression in the mid gut has been reported [21]. *SCARB1 *belongs to the ATP-binding cassette (ABC) transporter super-family, and in addition to lipoprotein, cell culture ligand binding studies show that SCARB1 recognizes and binds a diverse set of ligands with low substrate specificity, thus mediating the transport of many lipophilic substances [22,23]. The first evidence that SCARB1 was important for carotenoid transport was demonstrated in Drosophila, where a gene encoding a *SCARB1 *homologous protein is essential for the cellular uptake of carotenoids in this species [24]. It was later shown that SCARB1 facilitated the absorption of dietary β-carotene in mice [24,25], and likewise in the human intestinal derived cell line Caco-2 cells [26,27]. Recently, a study in *Bombyx mori*, identified a transmembrane protein Cameo2, homologous to the mammalian *SCARB1*, as a specific carotenoid transporter in the silk worm. This species represents a model system for selective carotenoid transport due to the presence of several genetic mutants with defects in parts of this pathway that result in altered cocoon pigmentation. In mutant larvae, Cameo2 expression was strongly repressed in the silk gland, resulting in colourless silk glands and white cocoons. The authors propose that selective delivery of lutein to specific tissues requires the combination of Cameo2 as a transmembrane receptor on the surface of the cell and an intracellular carotenoid binding protein (CBP) [28].

The role of SCARB1 in carotenoid transport in homologous systems prompted us to investigate if *SCARB1 *has a role in flesh pigmentation in Atlantic salmon. A resource population derived from an ancient land-locked population and a commercial production strain of Atlantic salmon in Norway, with considerable phenotypic variation for this trait, were used as a source to screen for genetic variation and to map *SCARB1-2 *to the Atlantic salmon genome.

## Results

### Identification of genetic variation in *SCARB1-2*

The founder parents of the QTL-mapping population (P0 SALBANK) were screened for genetic variation within the non-coding parts of Atlantic salmon *SCARB1 *[Genbank: DQ914655.1]. Intron-exon junctions were deduced from comparative alignment to the human *SCARB1 *reference sequence [Genbank: NM_005505.4]. PCR-amplification of Atlantic salmon [Genbank: DQ914655.1] intron three, four, five and ten were performed using oligonucleotides as described in table 1 and revealed the presence of several amplicons in intron four, while no nucleotide variations were detected in the remaining introns. The intron four length polymorphisms comprise an constant amplicon of about 1.5 kb, present in all subjects, and one to two additional amplicons ranging from 619 to 763 bp. DNA-sequencing revealed that the constant 1.5 kb amplicon equals the previously reported Atlantic salmon *SCARB1 *[Genbank: DQ914655.1]. The lower size amplicons were highly similar in sequence to the Atlantic salmon *SCARB1*. Novel oligonucleotides generated from the lower size amplicon-sequences confirmed the same amplification patterns as initially observed.

**Table 1:** Oligonucleotides

Oligonucleotide combination	Oligonucleotide forward (5'-3')	Oligonucleotide reverse (5'-3')	Size (bp)
1: Intron 3	AGCGTCTTCAGAAGCAGAAC	CCACATCAGCTCTCCTACAG	350

2: Intron 4	CTACGACAGCAAGCTGGTGGACTT	GGTCAAGCCGTTCCATGAGTTCAC	600-700

3: Intron 5	CTCCCCAGTGTAACATGATCAACGG	CAGATTGTCTGCAGGGACAGAAGC	600

4: Intron 10	GGGAAGATCTCAGAGGTTGT	TACACCATCACCATTGGCAA	500

5: Exon 4 and 5	CTACGACAGCAAGCTGGTGGACTT	GATGTCATCTTCGCCGGTATGGATT	138

6: cDNA	CGTTGGACCAATCATTATCGACA	TCTGTAGTGTAGTAGTACAGCTCCCAGAG	1308

7: cDNA	ATGAATAAATCTAAATTAGCGATCGGA	TGTCATCTTCGCCGGTATGGATT	672

8: cDNA	TGATGGAGAACTTGCCGTTCC	CCCTGCAGCACTGGGCTAGACT	1054

Oligonucleotides used for PCR-amplification, QTL-mapping and real-time PCR of the two *SCARB1 *paralogs in Atlantic salmon.

### Identification of a novel paralog of *SCARB1, SCARB1-2*

**Figure 1:**
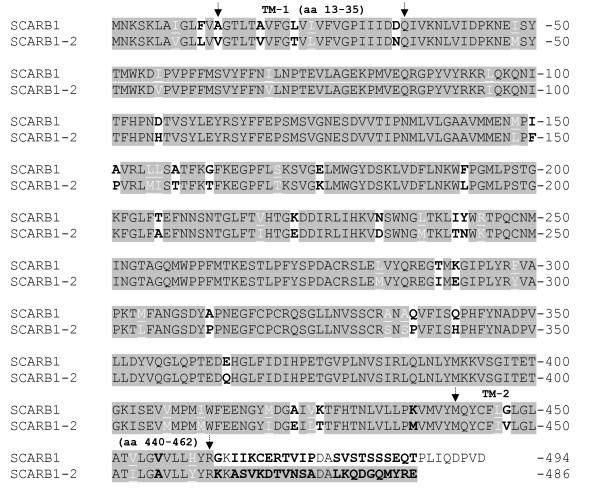
**The amino-acid (aa) sequence alignment of Atlantic salmon SCARB1 and the novel paralog, SCARB1-2**. The amino-acid (aa) sequence alignment of Atlantic salmon *SCARB1 *[NP_001117084] and *SCARB1-2 *[ADQ20116] shows that the sequences are 89% identical. 26 out of the 51 diverging aa are non-conservative (black letters in bold on white background), while the rest are conservative changes (white, underlined letters). Transmembrane domains (TM) are indicated by the vertical arrows.

The lower size amplicons of intron four potentially derive from a gene with high similarity to the coding sequence of *SCARB1*. In order to reveal the origin of these sequences, novel oligonucleotides were designed and used in RT-PCR, in combination with *SCARB1 *specific oligonucleotides. Further overlapping RT-PCR amplicons were generated using a combination of oligonucleotides highly similar to SCARB1, identified through the NCBI TRACE-archive sequences using Atlantic salmon *SCARB1*-sequence as a query [GenBank:DQ914655], in combination with the *SCARB1-2 *spesific sequence. In the end a cDNA-amplicon of 1309 bp, comprising n.p. 358-1666 of *SCARB1 *[GenBank:DQ914655], was obtained using oligonucleotide combination 6 (Table 1). A second RT-PCR using oligonucleotide combination 7 resulted in a PCR-amplicon comprising start codon at n.p. 281 to n.p. 953. Finally, a third amplicon was obtained using oligonucleotide combination 8 (Table 1), comprising n.p. 711-1765. The three above overlapping amplicons generated a transcript with an open reading frame extending from n.p. 281 to 1736 of *SCARB1 *[GenBank:DQ914655]. This full-length cDNA-sequence was blasted to the TRACE-archive and its sequence identity was confirmed for the *SCARB1-2 *cDNA at n.p.1-114, 417-833, 1001-1120, 1244-1343, and 1463-1612. The predicted protein shows 89% identity to Atlantic salmon SCARB1 [GenBank:DQ914655] when comparing the sequence from aa 1 to 462. The remainder of the protein, extending from aa 463 until stop codon at position 486, only shares 12% identity to SCARB1. SCARB1-2 is eight aa shorter compared to SCARB1 (Figure 1). The cDNA sequence and translated *SCARB1-2 *in Atlantic salmon is submitted to [GenBank: HQ403588].

### Linkage mapping of *SCARB1-2*

**Figure 2:**
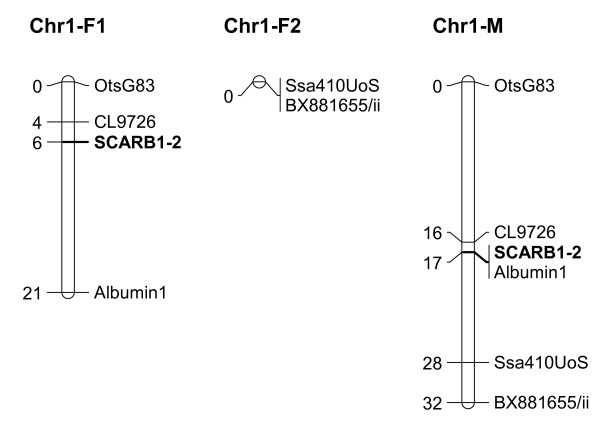
**Position of *SCARB1-2 *on the male and female chromosome 1 linkage maps**. The relative position of *SCARB1-2 *on the male(M) and female(F) chromosome 1 linkage maps used in this study. Chr1-F1 and Chr1-F2 represent two unlinked segments in the female map.

Genotyping of the six founding parents in the SALBANK population and the F2 progeny resulted in four amplifying alleles in the Bleke parents P11, P14 and P15 and three amplifying alleles in the commercial line parents P31, P34 and P35, ranging in size from 619 bp to 763 bp (table 2). Sequence-analysis revealed that the *SCARB1-2 *alleles comprise the zeste consensus motif [T/C]GAG[T/C][G/T]. The repetitive zeste-elements and their corresponding amplified alleles in the Bleke and the commercial Atlantic salmon parents are shown in table 2. Size determination of the *SCARB1-2 *alleles using fluorescently labelled oligonucleotides and capillary electrophoresis yielded no amplification products in parents P31, P34 and P35. These 'failed' amplifications were declared as 'null/null' genotypes, and when combined with the genotype patterns observed in the F2 progeny, it was possible to infer F1 parent genotypes with null alleles, and therefore Mendelian segregation in the F2 families. This strategy, however, meant that it was impossible to distinguish null/null genotypes from failed amplifications in F2 progeny. Nevertheless, based on high quality size standard profiles, and previous amplification success with other markers, null/null genotypes were inferred when no peaks were observed. *SCARB1-2 *mapped to chromosome 1 of the Atlantic salmon map [29] (Figure 2). Large differences in recombination rate were observed in the male and female maps, with the female map consisting of two unlinked segments (Chr1-F1 and Chr1-F2), while the male map had a higher level of recombination between marker OtsG83 and CL9726 than the female map.

**Table 2:** Genotypes of the founding parents.

Parents	Sequence	Genotype (bp)
P11 (Bleke)	**GGCGAGGG**TGAGTG**GGCGAGGG **TGAGTG	763, 763

P31 (AquaGen)	CGAGCG**AGAGGG**TGGGCG**AGAGGG**	null, null

P14 (Bleke)	not sequenced	619, 634

P34 (AquaGen)	CGAGCG**AGAGGG**TGAGTG**GG**	null, null

P15 (Bleke)	**GGCGAGGG **TGAGTG**GGCGAGGG **TGAGTG	623, 623

P35 (AquaGen)	CGAGCG**AGAGGG **TGAGTG**GG**	null, null

Genotypes of the founding parents (SALBANK) in the Bleke and Atlantic salmon commercial lines (AquaGen). Null genotypes represent non-amplifying alleles in the mapping population. The *SCARB1-2 *alleles, ranged in size from 623 to763 bp, and comprise the enhancer of zeste consensus motif (EZH2) [T/C]GAG[T/C][G/T] (underlined). The sequence of the repetitive EZH2-elements diverges for Bleke and the commercial Atlantic salmon.

### QTL (quantitative trait loci) analysis of *SCARB1-2 *alleles

**Figure 3:**
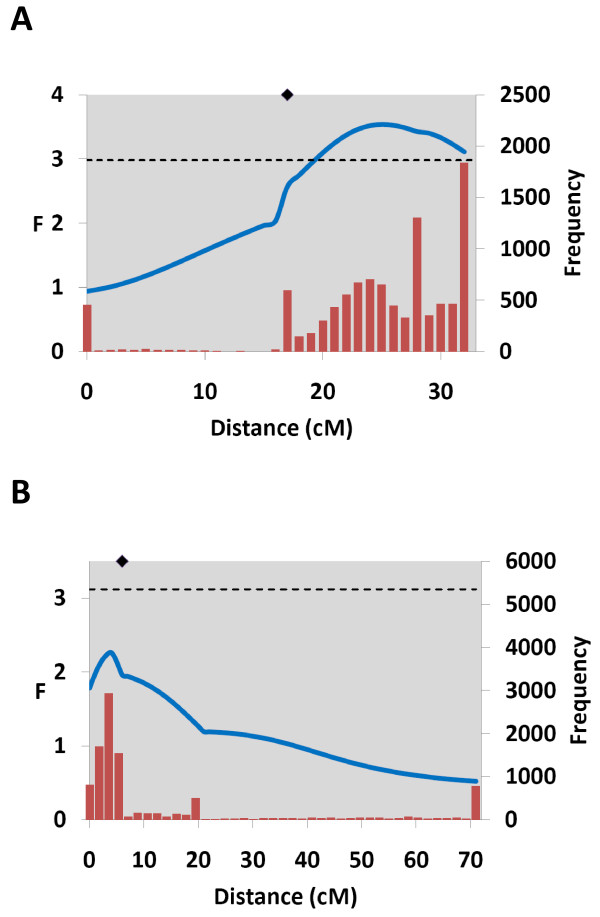
**QTL analysis of Atlantic salmon chromosome 1**. F-statistic profiles (blue lines) and QTL position bootstrap frequencies (red bars) for the male (A) and female (B) chromosome 1 linkage maps. Chromosome-wide significance thresholds are indicated by the horizontal broken lines. The position of *SCARB1-2 *is indicated by a black diamond on the upper x axis.

In the sire-based, across-family analysis, the QTL peak exceeded the chromosome-wide significance threshold (Figure 3), and the two parents M1 and M4 were found to be segregating for the QTL (table 3). In the dam-based across-family analysis, the overall F-value of 2.26 was not significant (Figure 3). However, parent F4 demonstrated strong evidence for QTL segregation, with an absolute t-value of 3.0, and significant QTL linkage was found when this parent was analysed individually (data not shown). In both sire and dam based analyses, the 95% confidence interval for the QTL position covered the entire linkage group, however the actual QTL peaks were 9 cM from *SCARB1-2 *in the male analysis and 2 cM from *SCARB1-2 *in the female analysis. After correction for an approximate selective genotyping fraction of 0.5 (averaged over families), the QTL explained 4.4% of the phenotypic variation for flesh colour. In family four, both parents appeared to be segregating for the QTL (table 4). The flesh colour averages for the different progeny genotypes (representing *QQ, Qq *and *qq *QTL allele combinations), indicated that the QTL alleles conferring redder flesh were inherited from both the Bleke and commercial founding parents in the two F1 parents.

**Table 3:** QTL segregating parents and estimates of QTL effects.

Parent	Estimate	SE	ABS(t)
**M1**	**0.89**	**0.37**	**2.4**

M2	0.53	0.37	1.4

M3	0.18	0.44	0.4

**M4**	**1.02**	**0.41**	**2.5**

F1	0.01	0.37	0.0

F2	0.06	0.37	0.2

F3	0.04	0.42	0.1

**F4**	**1.10**	**0.37**	**3.0**

QTL effect estimates and absolute t-values for the eight parents analysed (segregating parents in bold).

**Table 4:** Average SalmoFan scores for progeny genotype groups in the family with two QTL heterozygous parents.

Genotype	623 (Q)	null (q)
**634 (q)**	24.3 (Qq)	23.5 (qq)

**null (Q)**	26.5 (QQ)	24.8 (Qq)

Average SalmoFan values (uncorrected for body weight) for the different progeny genotype groups at *SCARB1-2 *in the family where both parents were QTL heterozygous. The inferred QTL allele is shown next to the *SCARB1-2 *marker allele for parents F4 (side) and M4 (top).

### *SCARB1 *and *SCARB1-2 *mRNA in Atlantic salmon tissues

**Figure 4:**
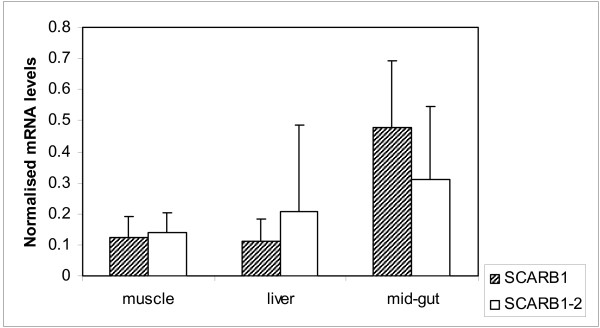
**Normalised expression of *SCARB1 *and *SCARB1-2 *transcripts in Atlantic salmon tissues**. Real-time PCR analyses showing the normalised *SCARB1 *and *SCARB1-2 *mRNA levels in Atlantic salmon muscle, liver and mid gut (n = 7-10), calculated by the 2^-ΔΔCt ^method and adjusted for PCR efficiencies. When comparing the gene expression in mid gut, muscle and liver for each paralog using one-way ANOVA, *SCARB1 *was found to be significantly increased in midgut compared to muscle and liver (P < 0.01). Average values of three independent qPCR runs were used for the calculation.

The expression pattern of *SCARB1 *and *SCARB1-2 *transcripts in liver, mid gut and muscle of commercial Atlantic salmon slaughtered prior to sexual maturity (NIVA fish) was investigated by real-time PCR. We chose the NIVA-fish as a reference population due to lack of tissues for RNA-extraction from the QTL-mapping population. Pair wise comparisons of the gene expression for each of the two paralogs revealed a five-fold increased expression of *SCARB1 *mRNA in mid gut, compared to that in muscle and liver (p < 0.01) (Figure 4). In contrast, the *SCARB1-2 *mRNA was about equally expressed in liver, muscle and mid gut (Figure 4). The Ct values in the real-time PCR analysis were about a ten-fold lower for *SCARB1-2 *mRNA, compared to *SCARB1*, and demonstrate an overall increased mRNA expression of *SCARB1*, compared to that of *SCARB1-2*.

## Discussion

The founding parents of the SALBANK resource population were screened for genetic variation in *SCARB1 *and revealed the presence of genetic variation in an adjacent novel paralog, which we named *SCARB1-2*. A total of seven different *SCARB1-2 *alleles were identified in the founder individuals, and the alleles diverged for the Bleke-and the commercial Atlantic salmon line as shown in table 2. Baranski *et al*. [30] found suggestive evidence (chromosome-wide significance) for a QTL with relatively large allele substitution effects in individual parents on this linkage group, and the addition of the *SCARB1-2 *marker data provided evidence for QTL segregation in a third parent, through increasing the information content in this family. Given that the QTL peak in both the sire and dam analyses is less than 10 cM from the position of *SCARB1-2 *(only 2 cM in the dam analysis) (Figure 3), *SCARB1-2 *represents a putative candidate gene for this QTL-region, however the large confidence intervals around the QTL peak imply that numerous other genes in this region could underly the QTL. Indeed, this large confidence interval is also reflected in the different positions of the QTL peaks in the two sexes, which can be explained by the substantial map difference between the sexes, and the differing levels of marker and QTL informativeness in the F1 parents. The tracing of the 'positive' allele to both the commercial and landlocked lines provides evidence for the heterozygosity of this QTL within the lines. This is not particularly surprising given the lines are outbred, and information on the carotenoid uptake of pure Bleke fish in an environment with ample pigment 'available' in the feed would be valuable information in further characterising the effect of environment versus genetics for this trait. Baranski *et al*. [30] also reported numerous other suggestive and significant QTL affecting this trait, suggesting a polygenic effect where genetic variation at other loci contributes substantially to the phenotypic variance observed.

Comparing the sequence identity of *SCARB1 *from aa position 1 to 462 to that of *SCARB1-2*, revealed an overall 89% sequence identity. Half of the diverging aa are of similar, or conservative character, while the remaining half is of dissimilar, or non-conservative character. The most diverging C-terminal region extends from aa 463 to the stop codon at aa 486. This C-terminal region comprises the cytoplasmic tail of the protein which has been shown to interact with proteins that can modulate localisation, stability and function of *SCARB1 *[31]. The relatively high degree of non-synonymous aa-substitutions, particularly in the C-terminal cytoplasmic tail, suggests that the two paralogs diverge in function. It has previously been hypothesised that flesh pigmentation is linked to the presence of a duplicated genome, as pigmentation appears to be found only in the genera *Salmo*, *Oncorhyncus*, *Salvelinus *and *Hucho*, those exhibiting partly tetrasomic inheritance [5]. Thus, it is possible that the genetic basis causing variation in flesh colour may be partly due to a duplicated gene like *SCARB1-2*.

Immunochemical methods have shown that *SCARB1 *is most highly expressed in adult mammalian tissues that are the principal sites of selective lipid uptake in vitro, i.e., the intestine, liver and steroidgenic tissues [32]. Studies in *SCARB1 *deficient mice and cell lines provide evidence for an involvement of SCARB1 in intestinal carotenoid absorption [25,26,33]. We found the most prominent expression of *SCARB1 *mRNA in the mid gut consistent with previous report on *SCARB1 *mRNA expression in Atlantic salmon [21]. The mid gut has been shown to be the main site of fat digestion and absorption in Atlantic salmon [33,34]. In skeletal muscle and liver the *SCARB1-1 *mRNA was reduced with a five-fold, compared to its expression in the intestine in Atlantic salmon (Figure 4). Interestingly, the *SCARB1-2 *mRNA level in muscle equals its expression in liver and mid gut, but is expressed at a considerably lower level than *SCARB1 *mRNA.

The different *SCARB1-2 *alleles, ranging in size from 619 to 763 bp, comprise repetitive sequences consisting of distinct enhancer of zeste motifs (EZH2) with the consensus sequence (T/C/g)GAGTG(A/G/c), recognized by the sequence-specific DNA-binding protein zeste. Zeste expression is developmentally regulated in skeletal muscle [35], and it has been shown that EZH2 is involved in controlling muscle gene expression and differentiation through removal of an actively suppressing HKMT protein complex containing Polycomb EZH2, and the subsequent engagement of positive transcriptional regulators characteristic for the activation of muscle gene expression [36]. It can be speculated that distinct *SCARB1-2 *alleles comprising these regulatory motifs may differently affect *SCARB1-2 *mRNA expression, and in turn, the cellular uptake of astaxanthin. However, the mapping results from this study are insufficient to confirm such an association, and further studies are needed to elucidate the effect of these regulatory motifs on the gene expression.

## Conclusions

Flesh pigmentation is an important quality trait in commercial Atlantic salmon that could be improved through elucidation of underlying genetic polymorphisms associated with the trait. SCARB1 has a key role in the cellular uptake of carotenoids in homologous systems. We here present a novel paralog of *SCARB1*, *SCARB1-2 *and identify alleles comprising repetitive enhancer of zeste motifs. These alleles were used to map *SCARB1-2 *to Atlantic salmon chromosome 1, a region containing a suggestive QTL for flesh colour based on a previous study. Inclusion of the *SCARB1-2 *marker strengthened the evidence for the presence of this QTL and is a potential candidate gene, however studies using a higher density marker coverage and larger experimental population will be needed to confirm association of the reported polymorphisms to flesh pigmentation over the large number of other genes in this chromosomal region. Gene expression analysis showed that *SCARB1-2 *mRNA was equally expressed in Atlantic salmon muscle, liver and mid gut, while *SCARB1 *mRNA was more prominent in the mid gut. Ultimately, further understanding of the molecular mechanisms involved in flesh pigmentation of salmonids will have direct benefits for genetic improvement of this trait and add valuable knowledge to our understanding of its evolutionary history.

## Methods

### Mapping population

The mapping population consisted of four F2 families that originated from a cross between two divergent populations of Atlantic salmon in Norway, the 'ancient', landlocked Byglands Bleke population and a commercial selected line, a resource population known as 'SALBANK'. These two populations are highly divergent for a number of traits, including flesh colour and growth, and the F2 population was created as a mapping resource with an increased likelihood of segregation at QTL affecting these traits. The details of the pedigree, phenotypic recording and sampling procedures can be found in Baranski et al. [30]. Fifty progeny from each extreme of the flesh colour distribution, measured visually using the Roche SalmoFan, were selected from three F2 families (8B, 9B and 10B), and all 76 progeny from a fourth family (10A) were selected for genotyping. Due to differences in progeny numbers between the families, this represented selective genotyping fractions (both extremes) of 44%, 35%, 35% and 100% respectively for the four families.

### Atlantic salmon tissues (NIVA fish)

The NIVA-fish served as a reference population (normal breeding population) since we did not have tissues for RNA from the actual QTL-mapping population. Tissues for total RNA-isolation were collected from Atlantic salmon with an average weight of 1000 grams (before sexual maturation), kept in tanks at 12°C at NIVAs Research station, Drøbak, Norway. Muscle, liver and mid gut were collected from ten fish, and immediately stored on liquid nitrogen and subsequently transferred to -80°C until RNA-extraction.

### DNA extraction

Genomic DNA was isolated from about 20 mg muscle of Atlantic salmon, using either MagAttract DNA M48 Tissue kit on the Bio-Robot M48 (Qiagen, Hilden, Germany) or DNeasy 96 protocol (Qiagen, Hilden, Germany). Prior to isolation, muscle samples were lysed in Proteinase K at 56°C over night according to the manufacturer's protocol. Insoluble materials after lysis were removed by centrifugation (300 × *g*, 1 minute). DNA was extracted according to manufacturer's instruction with the inclusion of *RNaseA *(0.1 mg/sample RNase A R5503 (Sigma-Aldrich, St. Louis, MO, USA)), for 30 minutes at room temperature.

### Oligonucleotide design

Oligonucleotides used for PCR-amplification of intron sequences were designed based on Atlantic salmon *SCARB1 *[Genbank:DQ914655.1] using Vector NTI Advance (Invitrogen, Carlsbad, CA, USA). Putative exon-intron junctions for intron three, four, five and ten were identified by comparative sequence alignment to the human *SCARB1 *reference sequence [Genbank: NM_005505]. For real-time PCR analysis oligonucleotides were designed to span one intronic sequence in order to keep control of any potential genomic contamination in the RNA samples.

### PCR

PCR-amplification was carried out using 0.4 μM oligo-nucleotides, 0.05 U/μl Taq Gold, 200 μM dNTPs and 1 × reaction buffer containing 15 mM MgCl_2 _(Applied Biosystems, Foster City, CA, USA). After 10 minutes of initial denaturation at 95°C, 40 cycles of amplification at 95°C for 30 seconds, 54-60°C for 30 seconds, 72°C for 30-60 seconds was followed by a final extension of 7 minutes at 72°C. The PCR-product was fractionated on a 2% agarose gel containing ethidium-bromide and visualised by UV-transiluminator 2000 (BIORAD).

### DNA sequence analysis

Prior to sequencing, the PCR-products were purified using Montage PCR Genomics cleanup kits (Millipore, Billerica, MA, USA), according to the manufacturer's protocol. DNA-sequencing was performed using the BigDye Terminator version 3.1 cycle sequencing kit according to the manufacturer's protocol with the following thermocycling conditions: 96°C 1 minute, 25 cycles at 96°C for 45 seconds, 50°C for 45 seconds and 60°C for 4 minutes. The products were purified with Montage-SEQ96 cleanup kits (Millipore, Billerica, MA, USA) and sequencing was performed on a 3730 DNA Analyser (Applied Biosystems, Foster City, CA, USA).

### RNA-isolation and cDNA-synthesis

Total RNA was isolated using a combined protocol with Trizol and RNeasy mini kit, and DNAse-treated using the RNase-free DNase set as described by the manufacturer (Invitrogen, Carlsbad, CA, USA). RNA quantity was measured using the NanoDrop ND-1000 Spectrophotometer (NanoDrop Technologies, Wilmington, DE, USA) and quality was examined by the 28S:18S rRNA ratio using the RNA 6000 Nano LabChip^® ^Kit on 2100 Bioanalyser (Agilent Technologies, Santa Clara, CA, USA). First strand cDNA synthesis was performed using SuperScript™-II Rnase H-Reverse Transcriptase (Invitrogen, Carlsbad, CA, USA) and oligo-dT oligonucleotide T270 (5'-GACTCGAGTCGACATCGATTTTTTTTTT-TTTTTTT-3'). 0.25 μg of total RNA was used as template for cDNA-synthesis, all RNA was standardised to the same concentration prior to cDNA-synthesis.

### Quantitative Real-Time PCR

Real-Time PCR was conducted using LC480 (Roche) and gene-specific oligonucleotides using oligonucleotide combination 2 and 5, for *SCARB1 *and *SCARB1-2*, respectively (table 1). Oligonucleotide and cDNA-concentration were optimised to obtain the lowest possible Ct-value. 0.4-1.25 μM of each oligonucleotide, 2 μl of 2 × SYBR Green PCR Mastermix (Applied Biosystems, Foster City, CA, USA) and the cDNA-template were mixed in a total volume of 12 μl. A two-step PCR was run for 45 cycles (10 s. at 95°C, 30 s. at 65-67°C) with an initial denaturation of 10 min. at 95°C. Specificity of the PCR-products was verified by agarose gel-electrophoresis and amplicon sequencing. Standard curves for each oligonucleotide pair were generated by serial dilution (1, 1:2, 1:10) of cDNA consisting of a pool of the representative samples, and PCR efficiencies (E) were calculated according to the formula E = 10^(-1/slope(a)) ^[37]. E-values was 0.7 for *SCARB1 *and for *SCARB1-2*, and comprise Ct-values ranging from 21 to 27, and from 30 to 34, respectively. Relative mRNA expression was calculated by the 2^-ΔΔCt ^method [38] adjusted for PCR efficiencies. 18S was used as a reference gene. Two independent cDNA-syntheses of each sample were performed. Two independent PCR reactions were run for each cDNA synthesis with duplicate samples within each PCR.

### Statistics

Statistical analyses of the qPCR results were carried out using a GenEx software package (MultiD Analyses AB, Sweden). The samples were already normalized to reference gene (18S) and PCR efficiency when imported into GenEx. The samples were normalized for technical replicates (and missing data), and calculated using the average of three independent qPCR runs. One-way ANOVA, with post test all pairwise comparisons (Tukey-Kramer's) was used to compare the gene expression in mid gut, muscle and liver, for each of the two paralogs.

### Linkage mapping and QTL analysis

Genotyping of the *SCARB1-2 *locus was carried out on the 'SALBANK' founder parents and F2 progeny with thermocycling conditions 95°C 10 min., 40 cycles of 95°C 30 sec., 60°C 30 sec., 72°C 30 sec., and 72°C 10 min., using oligonucleotide combination 5 shown in table 1. The lengths of the fluorescent PCR products were determined relative to the LIZ1200 size standard (Applied Biosystems, Foster City, CA, USA) on a 3730 DNA Analyzer (Applied Biosystems), using GeneMapper 4.0 (Applied Biosystems) software for allele calls. Genotypes of the F1 parents were inferred from the grandparent and offspring genotypes. The *SCARB1-2 *genotype data was added to the microsatellite data previously genotyped for these individuals [30], and linkage analysis was performed in Joinmap 3.0 using a minimum LOD score of 3.0 in order to map the *SCARB1-2 *locus to the Atlantic salmon linkage map. In order to evaluate QTL linkage with the hypothesis that a QTL peak is located at this marker position, half-sib regression interval mapping for the linkage group containing the *SCARB1-2 *locus was carried out in GridQTL at 1 cM intervals [39], with sex fitted as a fixed effect and body weight as a covariate due to a strong positive phenotypic correlation between body weight and colour. Separate male and female analyses were performed due to the map differences observed in the sexes. 50 cM distance was inserted between the unlinked pair of markers in the female map and marker *Albumin1 *in the first group. Significance thresholds were estimated after 10,000 chromosome-wide permutation tests. Parents segregating for the QTL were identified based on their individual t-values in the GridQTL analysis. Confidence intervals (CI) were estimated for the QTL using the bootstrap method and 10,000 iterations [40]. The proportion of phenotypic variance explained by the QTL using the half-sib model was calculated as (2*(1-MS_full _/MS_reduced_)^sire^) + (1-MS_full_/MS_reduced_)^dam^) [41].

## Abbreviations

aa: amino-acids; bp: base pairs; cM: centi Morgan; EZH2: enhancer of zeste motifs; kb: kilobases; n.p.: nucleotide position; QTL: quantitative trait loci; RT-PCR: reverse-transcriptase polymerase chain reaction; SCARB1: scavenger receptor class B, member 1, paralog 1; SCARB1-2: scavenger receptor class B, member 1, paralog 2; SNP: single nucleotide polymorphism

## Authors' contributions

HS performed the design of the study, carried out the molecular genetic studies and drafted the manuscript. HH performed parts of the molecular genetic studies and helped to draft the manuscript. MB performed the linkage mapping, QTL analysis and helped to draft the manuscript. DIV and SWO conceived the study and participated in its design and coordination and helped to draft the manuscript. All authors have read and approved the final manuscript.
